# Long-Lasting Immune Responses 4 Years after GAD-Alum Treatment in Children with Type 1 Diabetes

**DOI:** 10.1371/journal.pone.0029008

**Published:** 2011-12-12

**Authors:** Stina Axelsson, Mikael Chéramy, Maria Hjorth, Mikael Pihl, Linda Åkerman, Emanuela Martinuzzi, Roberto Mallone, Johnny Ludvigsson, Rosaura Casas

**Affiliations:** 1 Division of Paediatrics, Department of Clinical and Experimental Medicine, Faculty of Health Sciences, Linköping University, Linköping, Sweden; 2 INSERM, U986, DeAR Lab Avenir, Saint Vincent de Paul Hospital, Paris, France; 3 Université Paris Descartes, Sorbonne Paris Cité, Faculté de Médecine, Paris, France; 4 Assistance Publique – Hopitaux de Paris, Hôpital Cochin et Hôtel Dieu, Service de Diabétologie, Paris, France; La Jolla Institute of Allergy and Immunology, United States of America

## Abstract

A phase II clinical trial with glutamic acid decarboxylase (GAD) 65 formulated with aluminium hydroxide (GAD-alum) has shown efficacy in preserving residual insulin secretion in children and adolescents with recent-onset type 1 diabetes (T1D). We have performed a 4-year follow-up study of 59 of the original 70 patients to investigate long-term cellular and humoral immune responses after GAD-alum-treatment. Peripheral blood mononuclear cells (PBMC) were stimulated *in vitro* with GAD_65_. Frequencies of naïve, central and effector memory CD4+ and CD8+ T cells were measured, together with cytokine secretion, proliferation, gene expression and serum GAD_65_ autoantibody (GADA) levels. We here show that GAD-alum-treated patients display increased memory T-cell frequencies and prompt T-cell activation upon *in vitro* stimulation with GAD_65_, but not with control antigens, compared with placebo subjects. GAD_65_-induced T-cell activation was accompanied by secretion of T helper (Th) 1, Th2 and T regulatory cytokines and by induction of T-cell inhibitory pathways. Moreover, post-treatment serum GADA titres remained persistently increased in the GAD-alum arm, but did not inhibit GAD_65_ enzymatic activity. In conclusion, memory T- and B-cell responses persist 4 years after GAD-alum-treatment. In parallel to a GAD_65_-induced T-cell activation, our results show induction of T-cell inhibitory pathways important for regulating the GAD_65_ immunity.

## Introduction

Type 1 diabetes (T1D) is an autoimmune disease caused by auto-reactive immune cells which destroy insulin-producing β-cells, eventually leading to complete insulin deficiency [1]. Since auto-reactive T cells play a key role in the pathogenesis of T1D, they are considered an attractive therapeutic target for immunomodulatory strategies aiming at preventing or delaying disease onset [2], [3]. Glutamic acid decarboxylase 65 (GAD_65_) is one of the major autoantigens targeted by self-reactive T cells in T1D [4], [5]. Despite recent setbacks in a phase II [6] and a phase III clinical trial (submitted manuscript) using GAD_65_ formulated with aluminium hydroxide (GAD-alum), we and others have previously shown preservation of residual insulin secretion by GAD-alum treatment, in clinical phase II trials involving recent-onset T1D children [7] and LADA patients [8]. In addition to the clinical efficacy, we have reported that GAD-alum induced an early T helper 2 (Th2)-associated immune deviation in response to GAD_65_ [9] along with the appearance of GAD_65_-specific CD4^+^CD25^high^FOXP3^+^ cells [10]. The treatment also enhanced GAD_65_ autoantibody (GADA) levels [7] with an increase in subclasses IgG3 and IgG4 and a reduction in IgG1 suggestive of Th2 deviation, while IA-2 autoantibodies remained unaffected [11]. Altogether, these data indicate that GAD-alum treatment induced transient Th2-deviated GAD_65_-specific T- and B-cell responses during the 30-month study period. We have performed a 4-year follow-up study including 59 of the original 70 patients to evaluate long-term efficacy and safety of GAD-alum intervention. No treatment-related adverse events were reported and fasting C**-**peptide remained better preserved relative to placebo in patients with <6 months T1D duration at baseline [12].

Generation of a memory cell pool is important in the acquisition of effective immune therapy, and is formed by clonal expansion and differentiation of antigen-specific lymphocytes that ultimately persist for a lifetime [13]. Thus, the analysis of antigen-specific memory responses may be useful to understand the duration and stability of GAD-alum-induced immune responses. The leukocyte common antigen isoforms CD45RA and CD45RO have long been used to identify human naïve and memory T cells [14]. Naïve cells also express high levels of the chemokine receptor CCR7, which is essential for lymphocyte migration to lymph nodes [15]. Memory T cells contain two subsets, CD45RA^-^CCR7+ central memory (T_CM_) and CD45RA^-^CCR7- effector memory (T_EM_) cells, characterized by distinct homing capacities and effector functions [15]. Upon re-stimulation, T_EM_ show a low threshold for activation and produce cytokines with rapid kinetics. Antigen re-challenge also initiates a memory Th-controlled memory B-cell response that promotes robust antibody production and enhancement of the antigen-specific memory B-cell compartment [16].

The aim of this study was to evaluate the long-term antigen-specific memory T- and B-cell responses in T1D children treated with GAD-alum. We here show that treated patients display sustained GADA levels, increased memory T-cell frequencies and prompt T-cell activation upon *in vitro* stimulation with GAD_65_, 4 years after GAD-alum intervention. In parallel to a GAD_65_-induced T-cell activation, our results show induction of T-cell inhibitory pathways important for regulating the GAD_65_ immunity.

## Materials and Methods

### Ethics Statement

This study was approved by the Research Ethics Committee at the Faculty of Health Sciences, Linköping University, Sweden. Written informed consent was obtained from all patients, and for those <18 years old also their parents, in accordance with the Declaration of Helsinki.

### Subjects

The design and characteristics of the trial have previously been described [7]. Briefly, 70 T1D children between 10 and 18 years of age with less than 18 months of disease duration were recruited at 8 Swedish paediatric centres. All participants had a fasting serum C-peptide level above 0.1 nmol/l and detectable GADA at inclusion. Patients were randomized to subcutaneous injections of 20 µg GAD-alum (Diamyd^®^, Diamyd Medical; n = 35) or placebo (alum only; n = 35) at day 0 and a booster injection 4 weeks later in a double blind setting. After 4 years, patients and their parents were asked whether they were willing to participate in a follow-up study. Fifty-nine patients agreed to participate, of whom 29 had been treated with GAD-alum and 30 had received placebo.

### Isolation of PBMC

PBMC were isolated from sodium-heparinised venous fasting blood samples as described previously [9], and immediately stimulated *in vitro* for Luminex cytokine assay, PCR array and flow cytometry analyse. Remaining PBMC were cryopreserved in aliquots and used for T-cell enzyme-linked immunospot (ELISpot) and proliferation assays. It was not possible to perform all the different laboratory analyses on each study participant, due to the limited sample size. All laboratory work was performed in a blinded manner.

### Flow cytometry analysis

Staining of PBMC from GAD-alum- (n = 20) and placebo-treated patients (n = 23) was performed as previously described [17]. Briefly, PBMC were cultured for 7 days in AIM-V medium (Invitrogen) with or without 5 µg/ml of GAD_65_ (Diamyd Medical). After incubation, 10^6^ PBMC were stained with Alexa-700-conjugated anti-CD3, allophycocyanin (APC)-Cy7-conjugated anti-CD4, phycoerythrin (PE)-Cy7-conjugated anti-CD8, PE-Cy5-conjugated anti-CD45RA (BD Biosciences) and PE-conjugated anti-CCR7 (R&D Systems). Isotype controls (BD Biosciences) were included to estimate the amount of non-specific binding. Flow cytometry was performed with a BD FACSAria, and data analyzed in blind using Kaluza version 1.1 (Beckman Coulter). Lymphocytes were gated by forward (FSC) and side scatter (SSC) and the CD3+ events were plotted against side scatter to identify T cells.

### Gene expression analysis by quantitative Real-Time PCR array

Expression of 15 selected genes (Table 1) was analyzed using a customized Human Gene RT^2^ profiler™ PCR array (SABiosciences). PBMC were cultured for 24 h in AIM-V medium with or without 5 µg/ml of GAD_65_, and total RNA was isolated according to the RNeasy 96 vacuum/spin protocol (Qiagen) and quantified by optical density (OD) measurements at 260 nm. The purity of the RNA was ensured with an OD 260/280 ratio above 1.8, and RNA integrity was confirmed using Agilent 2100 bioanalyzer (Agilent Technologies). Each RNA sample (0.12 µg) was transcribed into PCR template with the RT^2^ First Strand Kit (SABiosciences). Templates were then combined with RT^2^ SYBR® Green/ROX™ qPCR Master Mix, and aliquots of 25 µl were loaded into each well containing the pre-dispensed gene-specific primer sets. ABI Prism 7900HT was employed for sequence detection, and sequence detection systems (SDS) version 2.3 (Applied Biosystems) was used to calculate the threshold cycle (Ct) values. An evaluation of the quality controls provided the relative levels of genomic DNA contamination and inhibition of either the reverse transcription or the PCR itself.

**Table 1:** Target genes included in the quantitative Real-Time PCR array.

Symbol	Accession	Description
*Cytokines*		
IL-2	NM_000586	Interleukin 2
IL-7	NM_000880	Interleukin 7
IL-15	NM_000585	Interleukin 15
TGF-β1	NM_000660	Transformig growthfactor, beta 1
*Cytokine receptors*		
IL-2RA (CD25)	NM_000417	Interleukin 2 receptor, alpha
IL-15RA (CD122)	NM_002189	Interleukin 15 receptor, alpha
*JAK/STATsignalling pathway*		
JAK3	NM_000215	Janus kinase 3
STAT5A	NM_003152	Signal Transducer andActivator of Transcription 5a
STAT5B	NM_012448	Signal Transducer andActivator of Transcription 5b
*T-cell regulators*		
FOXP3	NM_014009	Forkhead box P3
PDCD1	NM_005018	Programmed cell death 1
PD-L1 (CD274)	NM_014143	Programmed Death Ligand-1
BCL-2	NM_000633	B-cell lymphoma 2
*T-cell activation*		
CD69	NM_001781	CD69 molecule
*B-cell regulator*		
PRDM1	NM_182907	PR domain containing 1,with ZNF domain

Relative gene expression was calculated with the delta-delta Ct (ΔΔCt) method, using the normalized ΔCt value of each sample, calculated by subtracting the average Ct value of two housekeeping genes (GAPDH and HPRT1) from the Ct value of the gene of interest. The spontaneous Ct value was thereafter subtracted from the Ct value of the GAD_65_-stimulated sample. To calculate the ΔΔCt, the average ΔCt value of each gene in the placebo group was subtracted from the average Ct value of the corresponding gene in the GAD-alum group. The fold-change for each gene was calculated as 2^(−ΔΔCt)^.

### Lymphocyte proliferation assays

PBMC were re-suspended at 10^6^ cells/ml in AIM-V medium and incubated in triplicates (2×10^5^ cells/well) in round-bottom 96-well plates with 5 µg/ml GAD_65_, 10 µg/ml insulinoma antigen 2 (IA-2)_853-872_ peptide (ProImmune), 5 µg/ml tetanus toxoid (TTX; Statens Serum Institut), 5 µg/ml phytohaemagglutinin (PHA; Sigma) or no antigen. After 3 days, cells were pulsed for 18 h with 0.2 µCi of [^3^H] thymidine/well (Perkin Elmer), and thereafter harvested. Proliferation was recorded using a 1450 Wallac MicroBeta counter and expressed as stimulation index (SI), calculated as the median of triplicates in presence of stimulus divided by the median of triplicates with medium alone.

### Cytokine secretion assays

One million PBMC diluted in 1 ml AIM-V medium supplemented with 20 µM β-mercaptoethanol (Sigma) were cultured for 72 h in the presence of 5 µg/ml GAD_65_, 10 µg/ml IA-2_853–872_, 100 ng/ml TTX (Calbiochem) or in medium alone at 37°C in 5% CO_2_. The cytokines interleukin (IL)-1β, IL-2, IL-5, IL-7, IL-10, IL-13, IL-15, IL-17, tumour necrosis factor (TNF)-α and interferon (IFN)- γ were measured in cell culture supernatants using a Bio-Plex™ Human Cytokine Panel (Bio-Rad) according to the manufacturer's instructions as previously described [9]. The specific antigen-induced cytokine secretion was calculated by subtracting the spontaneous secretion (i.e. secretion from PBMC cultured in medium alone).

### Detection of antigen-specific T-cell responses by ELISpot

Detection of antigen-specific T-cell responses was performed with an accelerated co-cultured dendritic cell (acDC)-amplified ELISpot assay, as described [18]. Briefly, cryopreserved PBMC were thawed, washed twice in AIM-V medium and re-suspended at 10×10^6^/ml in AIM-V medium containing 1000 U/ml GM-CSF and 500 U/ml IL-4 (both from R&D). Cells were seeded at 10^6^/100 µl/well in flat-bottom 96-well plates and stimulated with 10 µg/ml GAD_65_, 40 µg/ml TTX (Statens Serum Institut) or no antigen at 37°C in 5% CO_2_. After 24 h, 100 µl AIM-V medium containing 100 U/ml TNF-α, 10 ng/ml IL-1β, 1 µM prostaglandin E2 and 0.5 ng/ml IL-7 was added to each well and cultured for another 24 h. Following this 48 h stimulation, non-adherent cells were washed, re-suspended in fresh AIM-V medium, seeded in quadruplicates at 1×10^5^ cells/well and incubated for 6 h in 96-well PVDF plates (Millipore) precoated with anti-IFN-γ or anti-IL-4 Abs (U-CyTech). Secretion of IFN-γ and IL-4 was visualized with a biotin-conjugated anti-IFN-γ or -IL-4 Ab (U-CyTech), alkaline phosphatase-conjugated ExtrAvidin and Sigmafast 5-bromo-4-chloro-3-indolyl phosphate/nitro blue tetrazolium (BCIP/NBT) tablets (both from Sigma), as described [19]. Spots were counted using a Bioreader 5000 Pro-SF (Bio-Sys). Means of quadruplicate wells were calculated and the results expressed as spot-forming cells (SFC)/10^6^ PBMC after background subtraction. The cut-off for a positive response was set at 3 SD above the average basal reactivity [19].

### Autoantibody and GADA IgG subclass analyses

Serum GADA and IA-2A titres were determined using a radio-binding assay employing ^35^S-labeled recombinant human GAD_65_ and IA-2, as previously described [11]. The GADA IgG1, 2, 3 and 4 subclasses were measured using a modification of the conventional GADA assay [11].

### GAD_65_ enzymatic activity assay

Recombinant human GAD_65_ enzymatic activity was measured in the presence of patient serum by a ^14^CO_2_-trapping method based on the enzymatic conversion of glutamate to GABA as previously described [11], and expressed as a percentage of the maximum GAD_65_ enzymatic activity. As GADA-positive serum from Stiff person syndrome (SPS) patients has been shown to inhibit this reaction [20], serum from one SPS patient was included in each assay as a positive control for inhibition.

### C-peptide

C-peptide levels were measured in serum samples with a time-resolved fluoroimmunoassay (AutoDELFIA^TM^ C-peptide kit, Wallac), described previously [7]. Stimulated C-peptide was measured during a mixed meal tolerance test (MMTT) in patients who had a maximal C-peptide response of >0.20 nmol/l at the 30-month follow-up, i.e. 21 GAD-alum-treated patients and 10 patients in the placebo group. Clinical effect of treatment was defined by changes in stimulated C-peptide measured as area under the curve (AUC) from baseline to 48 months.

### Statistical analysis

As the immunological markers were not normally distributed, non-parametric tests corrected for ties were used. Unpaired analyses were performed using the Mann-Whitney *U*-test, and correlations were analysed with Spearman's rank correlation coefficient test. Differences within groups were calculated by Wilcoxon signed rank test. A probability level of <0.05 was considered statistically significant. Calculations were performed using PASW statistics version 18 for Windows (SPSS Inc).

## Results

### GAD-alum-treated patients display increased memory CD4+ T-cell frequencies after *in vitro* GAD_65_ stimulation

**Figure 1:**
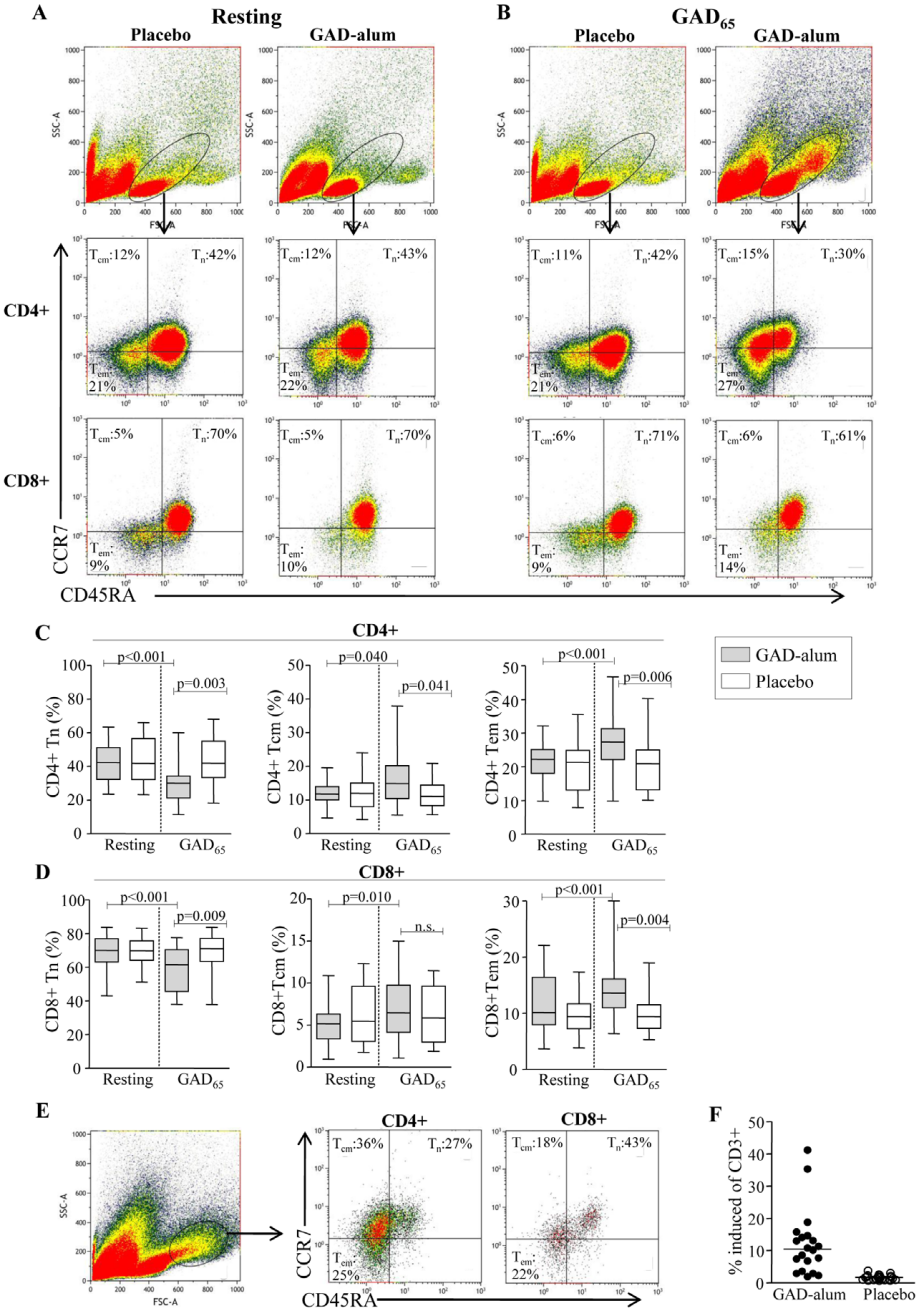
Frequencies of naïve, central memory and effector memory CD4+ and CD8+ T cells. **(A-B)**Representative flow cytometry analysis from one placebo- and one GAD-alum-treated-patient assessed in resting and GAD_65_-stimulated PBMC cultures. The gate was set to include both small and large lymphocytes. Median percentages of naïve (CD45RA^+^CCR7^+^;T_n_), central memory (CD45RA^-^CCR7^+^;T_CM_) and effector memory (CD45RA^-^CCR7 ^-^;T_EM_) CD4+ and CD8+ T cells are indicated in each plot. (**C**–**D**) Frequencies of naïve (T_n_), central memory (T_CM_) and effector memory (T_EM_) CD4+ and CD8+ T cells, assessed in resting and GAD_65_-stimulated PBMC cultures from GAD-alum- (n = 20) and placebo-treated patients (n = 23). Box plots represents median and range, significant differences are indicated as p-values. (**E**) Representative dot plot of the GAD_65_-induced cell subset with higher SSC and FSC evident only in GAD-alum-treated patients. Median percentages of naïve and memory CD4+ and CD8+ subsets are indicated in each plot. (**F**) Frequency of CD3+ cells that occupied the induced cell subset. Horizontal line represent median.

To test whether their frequencies were altered after GAD-alum-treatment, naïve and memory CD4+ and CD8+ subsets were analyzed in resting (Fig. 1A) and GAD_65_-stimulated (Fig. 1B) PBMC. The frequency of naïve (CD45RA^+^CCR7^+^), T_CM_ (CD45RA^-^CCR7^+^) and T_EM_ (CD45RA^-^CCR7^-^) CD4+ cells in resting cultures did not differ between the placebo and GAD-alum groups (Fig. 1C). When stimulated with GAD_65_, frequencies of T_CM_ (p = 0.040) and T_EM_ (p<0.001) increased whereas naïve cells decreased (p<0.001) in GAD-alum-treated patients, while the placebo group remained unaffected. The frequency of T_CM_ (p = 0.041) and T_EM_ (p = 0.006) was also significantly higher, and naïve cells lower (p<0.001) in the GAD-alum group compared to the placebo group.

Similarly, the frequency of naïve, T_CM_ and T_EM_ CD8+ cells in resting cultures did not differ between the placebo- and GAD-alum-treated patients (Fig. 1D). However, after GAD_65_-stimulation, the frequency of T_CM_ (p = 0.010) and T_EM_ (p<0.001) increased in GAD-alum-treated patients, whereas naïve CD8+ decreased (p<0.001). The proportion of T_EM_ was also significantly higher (p = 0.004), and naïve cells lower (p<0.001) compared to the placebo group, which remained unaffected upon GAD_65_-stimulation.

Induction of a cell subset with higher SSC and FSC was evident upon GAD_65_-stimulation only in GAD-alum treated patients (Fig. 1E–F). The majority of cells within this population were CD4+ with a memory phenotype.

### *In vitro* stimulation with GAD_65_ induces T-cell activation and proliferation in GAD-alum-treated patients

**Figure 2:**
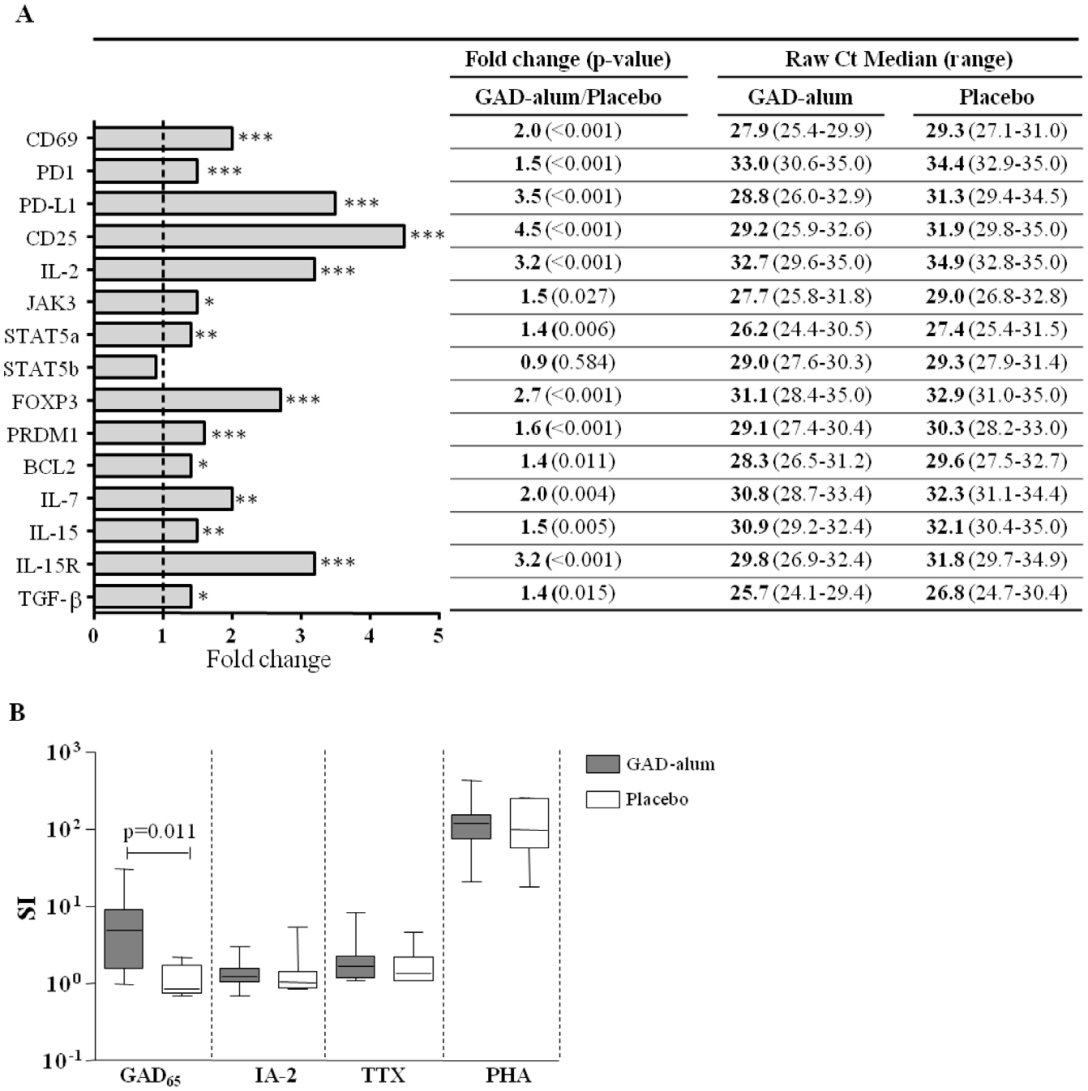
GAD_65_-induced gene expression and PBMC proliferation. (**A**) Fold change for 15 target genes between placebo (n = 19) and GAD-alum-treated (n = 18) patients. The ΔΔCt was calculated by subtraction of the average ΔCt value of each gene in the placebo group from the average Ct value of the corresponding gene in the GAD-alum group. The fold-change for each gene was calculated as 2^(−ΔΔCt)^. Raw threshold cycle (Ct) values (median and range) for the two groups are listed. (**B**) Proliferative responses (median and range) to GAD_65_, IA-2_853–872_, TTX and PHA in GAD-alum- (n = 10) and placebo-treated patients (n = 7). Proliferation is expressed as stimulation index (SI) calculated from the median of triplicates in the presence of stimulus divided by the median of triplicates with medium alone. Significant differences are indicated as p-values.

As memory T cells are characterized by a low activation threshold, we next analyzed the effect of antigen challenge on the induction of T-cell activation markers and proliferative responses. GAD_65_-induced gene expression of CD69, CD25 and PD-1 was up-regulated in patients treated with GAD-alum (n = 18) compared to placebo (n = 19) (2-, 4.5- and 1.5-fold, respectively; p<0.001; Fig. 2A). In addition, up-regulation of components of the IL-2 signalling pathway including IL-2 (3.2-fold; p<0.001), JAK3 (1.5-fold; p = 0.027) and STAT5a (1.4-fold; p = 0.006), together with the transcription factors FOXP3 (2.7-fold; p<0.001) and PRDM1 (1.6-fold; p<0.001), and the anti-apoptotic molecule BCL-2 (1.4-fold; p = 0.011) was detected upon GAD_65_-stimulation in GAD-alum- vs. placebo-treated patients. Furthermore, the PD-1 ligand (PD–L1) was also markedly up-regulated (3.5-fold; p<0.001) in GAD-alum-treated patients. Other up-regulated markers included IL-7 (2.0-fold; p = 0.004), IL-15 (1.5-fold; p = 0.005), IL-15 receptor (3.2-fold: p<0.001) and TGF-β (1.4-fold; p = 0.015), while STAT5b and the housekeeping genes GAPDH and HPRT1, to which all expression were normalized, were not significantly different between the two treatment arms.

Proliferative responses to GAD_65_ were also significantly higher in the GAD-alum-treated patients compared to placebo (p = 0.011; Fig. 2B). In contrast, proliferative responses to the control antigens IA-2_853-872_ (T1D-associated antigen), TTX (irrelevant control) and PHA (positive control) did not differ between GAD-alum- and placebo-treated patients.

### GAD_65_-stimulation of PBMC induces cytokine secretion in GAD-alum-treated patients

**Figure 3:**
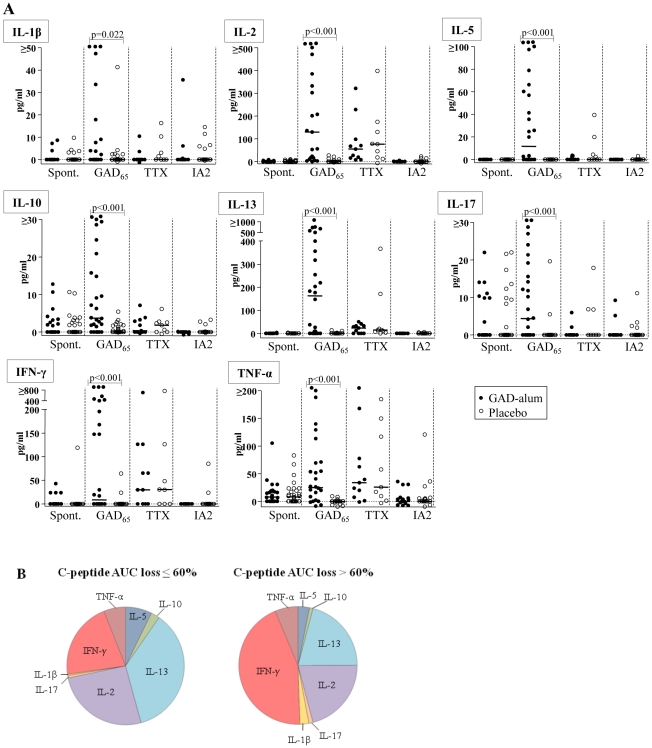
Cytokine secretion upon *in vitro* PBMC stimulation. (**A**) Spontaneous, and GAD_65_-, TTX- and IA-2_853–872_-induced IL-1β, IL-2, IL-5, IL-10, IL-13, IL-17, IFN-γ and TNF secretion (pg/ml), detected by Luminex in PBMC supernatants after 72 hour culture, from GAD-alum- (black circles; n = 28) and placebo-treated patients (white circles; n = 29). Antigen-induced cytokine secretion is given after subtraction of spontaneous secretion. Horizontal lines represent medians. Significant differences are indicated as p-values. (**B**) Cytokine profiles in GAD-alum patients with a loss of C-peptide AUC <60% (n = 5), or a loss of C-peptide AUC >60% (n = 16). Pie chart illustrates the relative contribution of each cytokine to the GAD_65_-induced secretion detected by Luminex.

Since memory T cells are capable of immediate effector cytokine production when stimulated *in vitro*, we sought to study the cytokine profile in PBMC supernatants after antigen challenge using a multiplex Luminex assay. When stimulated with GAD_65_, secretion of IL-1β, IL-2, IL-5, IL-10, IL-13, IL-17, IFN-γ and TNF-α was higher in PBMC from GAD-alum-treated patients compared to the placebo group (Fig. 3A). In contrast, spontaneous as well as TTX and IA-2_853–872_-induced secretion were similar in the two groups. Although the secreted levels of IL-7 and IL-15 were below the detection limit in the Luminex assay and although TGF-β was not available for multiplex testing, mRNA expression of these cytokines was up-regulated in PBMC from GAD-alum-treated patients re-challenged with GAD_65_, as shown in Fig. 2A.

In order to search for immune surrogate markers of clinical efficacy, we analyzed the association between cytokine secretion and β-cell function, as measured by stimulated C-peptide. No statistical significant associations were observed between cytokine production, or any other immune marker included in this study, and stimulated C-peptide. Still, to graphically illustrate the cytokine profile in relation to clinical efficacy, GAD-alum-treated patients were divided in two subgroups; patients with a loss of C-peptide AUC ≤60% (n = 5), and patients with a loss of AUC >60% (n = 16; Fig. 3B). The cytokine profile in patients with a loss of AUC ≤60% was characterized by a more pronounced GAD_65_-induced IL-5, IL-10, IL-13 and IL-2 secretion, whereas patients with a loss of AUC >60% had a more pronounced inflammatory profile characterized by IFN-γ, IL-1β and IL-17 secretion.

### GAD-alum-treated patients display higher numbers of IFN-γ- and IL-4-secreting T cells upon *in vitro* GAD_65_-stimulation

**Figure 4:**
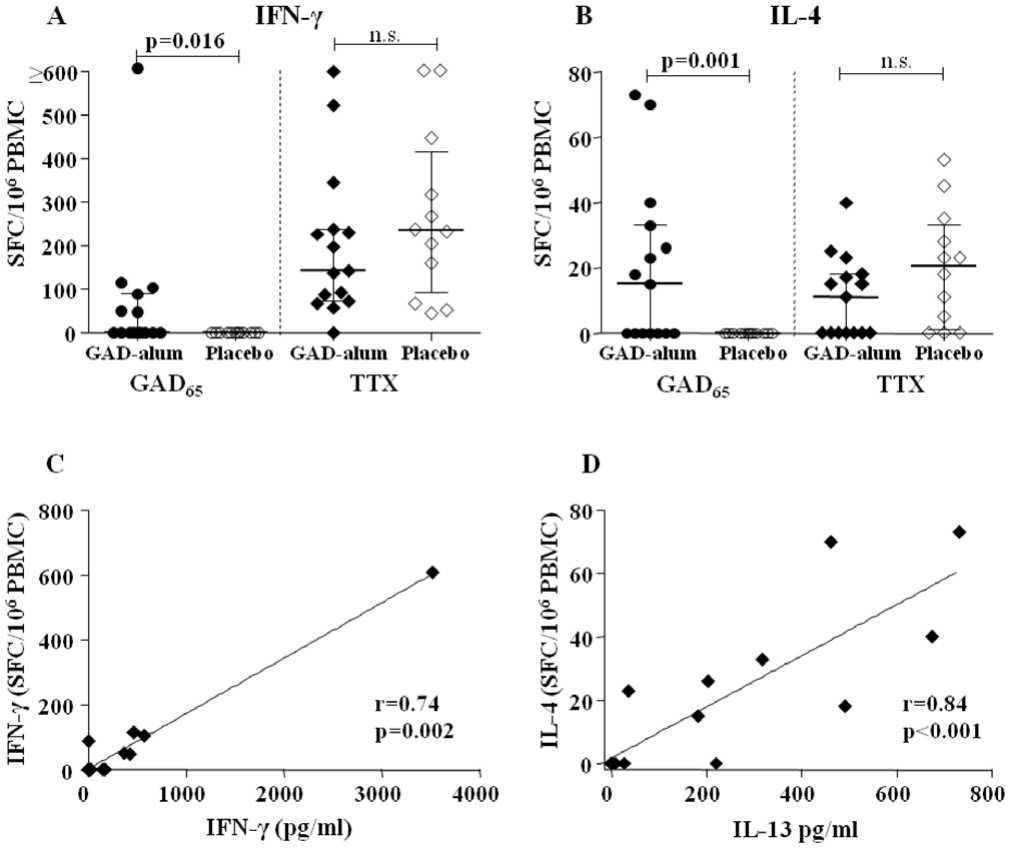
Quantification of antigen-specific T-cell responses by an acDC-amplified ELISpot assay. GAD_65_- and TTX-induced (**A**) IFN-γ and (**B**) IL-4 spot forming cells (SFC) in GAD-alum- (filled symbols, n = 15) and placebo-treated patients (open symbols, n = 12), expressed as SFC/10^6^ PBMC calculated from the mean of quadruplicates after background subtraction. Median and interquartile range is indicated. (**C**) Correlation between GAD_65_-induced IFN-γ SFC, detected by acDC ELISpot and IFN-γ secretion, detected by Luminex, in GAD-alum treated patients. (**D**) Correlation between GAD_65_-induced IL-4 SFC, detected by acDC ELISpot and IL-13 secretion, detected by Luminex, in GAD-alum-treated patients. Significant differences are indicated as p-values and correlation coefficient as r.

Quantification of antigen-specific T-cell responses was performed using an acDC-amplified ELISpot readout [18]. The GAD_65_-induced IFN-γ (p = 0.016) and IL-4 (p = 0.001) spot forming cells (SFC) were significantly increased in GAD-alum-treated patients compared to the placebo group (Fig. 4A-B), whereas TTX-induced SFC was similar in the two groups. Furthermore, GAD_65_-induced IFN-γ SFC correlated with GAD_65_-induced IFN-γ secretion (r = 0.74, p = 0.002; Fig. 4C), and IL-4 SPC with secretion of the Th2 cytokine IL-13 (r = 0.84, p<0.001; Fig. 4D), in the GAD-alum-treated patients.

### Sustained high serum GADA titres in GAD-alum-treated patients

**Figure 5:**
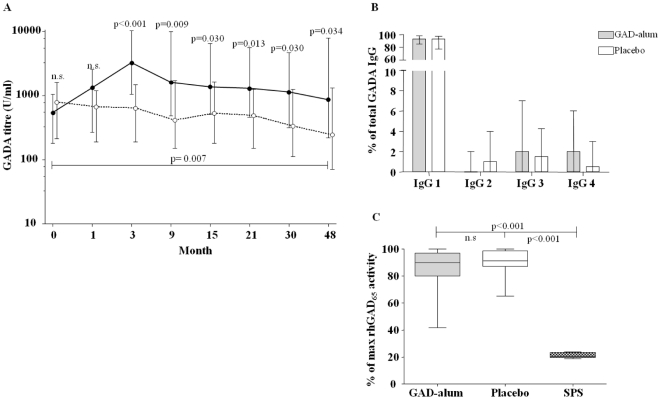
Serum GADA levels, GADA IgG1-4 subclass distribution and GAD_65_ enzymatic activity. (**A**) Serum GADA titres (U/ml; median and interquartile range) from baseline to 4 years, in GAD-alum treated patients (filled symbols, n = 29) and in placebo (open symbols, n = 30). (**B**) Serum GADA IgG 1–4 subclass distribution (median and range), expressed as a percentage of total GADA IgG in GAD-alum- and placebo groups. (**C**) Recombinant human GAD_65_ enzymatic activity measured in the presence of serum from GAD-alum- and placebo-treated patients. One SPS patient was included in each assay as a positive control for enzymatic inhibition. Results are reported as a percentage (median and range) of maximum GAD_65_ enzymatic activity. Significant differences are indicated as p-values.

Since autoantibody determination may be useful in assessing the long-lasting immunological impact of autoantigen treatment, we next analysed serum GADA titres. Our results show higher GADA levels in GAD-alum-treated patients compared to placebo, 4 years after treatment (p = 0.034; Fig. 5A). The GADA titres were also higher compared to baseline levels in the GAD-alum- (p = 0.007) but not in the placebo-treated group. In addition, IA-2A levels were determined in order to confirm that the persistent humoral response was antigen-specific. No difference between the two groups was observed (not shown). Further, the GADA IgG 1-4 subclass distribution was determined, as Th1- and Th2-cell cytokine production influence IgG-subclass switching [21], [22]. However, the subclass distribution did not differ between groups (Fig. 5B), nor did it differ compared to baseline (not shown).

High GADA titres are often found in SPS patients, raising concerns that therapies boosting GADA may have deleterious neurological effects. However, SPS is characterized by GADA which inhibit the GAD_65_ enzymatic activity [20], which is not the case in T1D. Therefore, we investigated whether serum containing high GADA titres generated by GAD-alum treatment, was inhibitory. The enzymatic activity did not differ when rhGAD_65_ was incubated with serum from GAD-alum- (median 90 %, range 42-100) and placebo-treated patients (median 91 %, range 65-100), but was significantly higher compared to GAD_65_ incubated with control serum from a SPS patient (median 20 %, range 19-24; p<0.001; Fig. 5C).

## Discussion

We have shown significant preservation of residual insulin secretion 4 years after GAD-alum treatment in T1D children and adolescents with <6 months T1D duration at inclusion, compared to placebo [12]. In the present study, we aimed to characterize the long-term antigen-specific memory T- and B-cell responses. Detection of antigen-specific memory cells *ex vivo* is a great challenge due to low frequencies. A previous study has demonstrated that only one in 30,000 or less CD4+ T-cells in peripheral blood from patients with recent-onset T1D is GAD_65_-specific [23], and activation and *in vitro* amplification of the GAD_65_-specific T-cells is crucial for detection. Our results show induction of T-cell subsets with a predominant memory phenotype upon *in vitro* GAD_65_-stimulation in PBMC from GAD-alum-treated patients. This suggests clonal expansion of the memory T-cell compartment upon antigen re-challenge, in parallel to the observed proportional reduction in naïve T-cell percentage.

When stimulated *in vitro*, memory T cells display low activation thresholds, immediate cytokine production and vigorous proliferation [13]. Our results show that the T-cell activation markers CD69, CD25 and PD-1 were all up-regulated in PBMC from GAD-alum-treated patients, and that proliferative T-cell responses to GAD_65_ together with GAD_65_-induced cytokine secretion were significantly higher compared to placebo, the latter confirming our previous findings [7], [9], [10]. Given that the cytokine- and proliferative responses elicited by various control antigens *in vitro* were similar between the two treatment groups, the effect of GAD-alum appears to be antigen-specific. This selective immune modulation might also be considered as an indication of safety, since it would be undesirable to non-specifically influence immune responses to unrelated antigens. Besides detecting cytokine secretion in PBMC, we quantified antigen-specific IFN-γ and IL-4 T-cell responses using an acDC assay. PBMC were selected on the basis of availability and thereby a limiting factor for including additional cytokines. In the assay, antigen and DC-activating agents rapidly induce, pulse and mature DCs, thus lining up the sequential steps of T-cell activation within 48 h and amplifying antigen-specific responses. The utility of these acDC-based assays for immune monitoring of vaccination trials has been previously demonstrated [18], [24]. The number of GAD_65_-induced IL-4 SFC, a cytokine difficult to detect with the Luminex assay, was significantly increased in the GAD-alum group compared to placebo. Further, IL-4 SFC correlated with IL-13 secretion, two Th2 cytokines with overlapping biological effects that share receptor components [25]. In parallel, the GAD_65_-induced IFN-γ SFC were also increased in the GAD-alum-treated patients, and correlated with the GAD_65_-induced IFN-γ secretion, supporting the reliability of the cytokine observations.

Proliferation of memory T cells can be driven not only by antigenic stimulation but also by cytokines. Here we show that gene expression of IL-7 and IL-15, two cytokines that are constitutively produced by a variety of cells and play an essential role for maintenance of both CD4+ and CD8+ T cells [13], was higher after GAD_65_-stimulation in the GAD-alum-treated group. In addition, IL-2, which is involved in long-term survival of antigen experienced CD4+ and regulatory T cells [26], [27], was also induced by GAD_65_-stimulation. Receptors for IL-2, IL-7 and IL-15 transmit signals mainly through STAT5, which is a critical factor for inducing and maintaining the expression of FOXP3 [28], and of the anti-apoptotic molecule Bcl-2 [29]. Up-regulation of the aforementioned cytokines and their receptors upon GAD_65_-stimulation, together with that of their associated signalling pathways and transcription factors suggests their involvement in the maintenance of a long-lasting GAD_65_-specific T-cell memory population. B-cell memory is characterized by persistent elevated specific antibody titres and generation of long-lived memory B cells [30]. Elevated GADA levels 4 years after GAD-alum-treatment, together with up-regulated PRDM1, a transcription factor essential for development of Ig-secreting cells and maintenance of long-lived plasma cells [31], suggests an induction of plasma cells continuously secreting GADA. PRDM1 is also expressed in effector and memory T cells [32], [33], and appears to have a role in Th2 cells by repressing Th1 genes [34].

The outcome of a T-cell response is shaped by the balance between co-stimulatory and co-inhibitory signals, which are often simultaneously provided to T cells by their surrounding cells. PD-1 is a member of the CD28 superfamily of immunoreceptors that is up-regulated following TCR stimulation [35], and interaction with its ligand PD-L1 inhibits T-cell effector functions [36]. Up-regulation of PD-1/PD-L1 in parallel to GAD_65_-induced T-cell activation and proliferation in the GAD-alum group demonstrates activation of co-inhibitory pathways important for regulating the immune balance.

Reliable biomarkers associated with therapeutic success following vaccination with β-cell antigens are still lacking. We have previously shown that, although GAD-alum-treatment induced a GAD_65_-specific cell population characterised by a broad cytokine profile [7], the response was preceded by an early Th2 immune deviation [9]. The cytokine profile observed in patients with better preserved C-peptide after 4 years, even though not statistically assured, may suggest that a beneficial clinical response might be associated with a persistent Th2/Treg-skewed GAD_65_-specific immune response. In a vaccination trial by Harrison and co-workers using intranasal insulin, immune responses were characterized by IFN-γ ELISpot and autoantibody measurements [24]. In contrast to our findings, antigen-specific IFN-γ and antibody responses decreased following treatment, suggesting that the therapeutic effect (or lack thereof) may be linked to different immunological mechanisms. The administration route and the use of alum adjuvant may be important factors in triggering these different mechanisms. Recently a phase II trial [6] and a European phase III trial (submitted manuscript) using GAD-alum have failed to reach their primary outcome. However, it cannot yet be excluded that treatment might be beneficial in certain patient subgroups. Thus, in order to make improvements in β-cell antigen treatment, alone or in combination with other therapies, it is of utmost importance to learn more about the immunological effects.

In conclusion, we here show persistent GAD_65_-specific cellular- and humoral immune responses 4 years after GAD-alum intervention in T1D children. Prompt re-activation of GAD_65_-reactive T cells upon *in vitro* antigen challenge was accompanied by secretion of Th1, Th2 and Treg cytokines and by induction of co-inhibitory pathways that may be of importance for regulating the GAD_65_ immunity.
